# Regulation of cell function and identity by cellular senescence

**DOI:** 10.1083/jcb.202401112

**Published:** 2024-06-12

**Authors:** Anda Huna, Amélie Massemin, Gabriela Makulyte, Jean-Michel Flaman, Nadine Martin, David Bernard

**Affiliations:** 1 Equipe Labellisée la Ligue Contre le Cancer, Centre de Recherche en Cancérologie de Lyon, Inserm U1052, CNRS UMR 5286, Université de Lyon, Centre Léon Bérard, Lyon, France

## Abstract

During aging and in some contexts, like embryonic development, wound healing, and diseases such as cancer, senescent cells accumulate and play a key role in different pathophysiological functions. A long-held belief was that cellular senescence decreased normal cell functions, given the loss of proliferation of senescent cells. This view radically changed following the discovery of the senescence-associated secretory phenotype (SASP), factors released by senescent cells into their microenvironment. There is now accumulating evidence that cellular senescence also promotes gain-of-function effects by establishing, reinforcing, or changing cell identity, which can have a beneficial or deleterious impact on pathophysiology. These effects may involve both proliferation arrest and autocrine SASP production, although they largely remain to be defined. Here, we provide a historical overview of the first studies on senescence and an insight into emerging trends regarding the effects of senescence on cell identity.

## Teaser

Huna et al. provide insights into the shifting perception of cellular senescence: from a mere cell proliferation arrest to a reinforcement or change of cell identity.

## Historical overview of cellular senescence: How effects of senescent cells were largely attributed to loss of cell proliferation and function

Cellular senescence was first described by Leonard Hayflick, who reported that fibroblasts in culture have a limited replicative potential (Hayflick and Moorhead, 1961). This process was then associated with telomere shortening (Harley et al., 1990) and termed replicative senescence. Later, stresses including oncogene activation, oxidants, or DNA damaging agents were shown to cause cells to enter a similar state of senescence independently of telomere shortening (Chen and Ames, 1994; Serrano et al., 1997). This senescence was termed premature senescence.

Senescence-associated proliferation arrest is induced by cyclin-dependent kinase inhibitors (CDKi), mainly the p53 target p21, inhibiting a broad spectrum of CDKs, and p16 inhibiting CDK4/6. In both cases, CDK inhibition leads to RB binding to the E2F transcription factor, which results in the repression of the E2F target genes that promote cell cycle progression (Stein et al., 1999). No specific senescence marker has so far been described, and the characterization of senescence thus relies on several features related to senescent cells, including increased expression of p21 or p16, decreased expression of the proliferation marker Ki67, and decreased BrdU incorporation in DNA, all used to document proliferation arrest (Gorgoulis et al., 2019), and senescence-associated-β-galactosidase staining (SA-β-Gal) (Dimri et al., 1995), which reflects increased lysosomal content.

**Figure 1.**
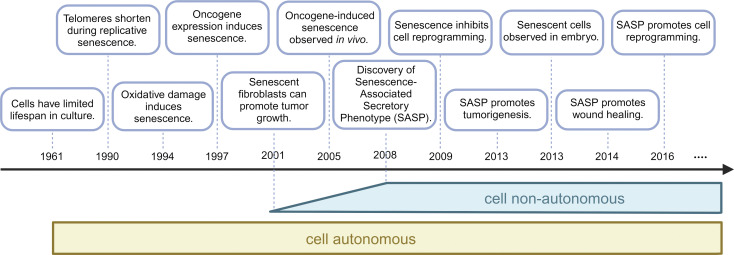
**Timeline of discoveries on the effects of senescent cells.** In this timeline, starting at the discovery of replicative senescence in 1961, selected milestones of understanding biology and function of senescent cells are shown. Discoveries in 2001 and 2008 of secreted factors of senescent cells mark the shift from considering effects of senescent cells only as cell autonomous to acknowledging non-autonomous effects of senescence.

From its discovery up to 2008, cellular senescence was primarily viewed as a cell state globally regulating proliferation-associated processes by irreversibly stopping cell proliferation, thus preventing cell immortality, blocking cancer development, and limiting organismal lifespan and globally regulating proliferation-associated processes (Fig. 1).

As such, cellular senescence is a critical antitumoral barrier in the initial steps of tumorigenesis as it blocks the proliferation of damaged cells. However, ∼90% of all cancers express telomerase and are therefore immortal and protected from telomere shortening–induced replicative senescence (Stewart and Weinberg, 2000; Kim et al., 1994). In addition, oncogene expression in normal cells induces a type of premature senescence named oncogene-induced senescence (OIS) (Serrano et al., 1997). Although the relevance and existence of cellular senescence in vivo was quite debated (Sherr and Depinho, 2000), in 2005, several articles demonstrated the accumulation of senescent cells in early human tumoral lesions and/or in response to different oncogenes in mouse models. For instance, in lymphoma cells, RAS activation leads to senescence, which depends on p53 and senescence-specific chromatin changes (methylation of histone H3 lysine 9), while inhibition of p53 and senescence leads to lymphoma (Braig et al., 2005). In addition, in human and mouse naevi, which are benign tumors harboring a BRAF mutation, which is a downstream effector of RAS, melanocytes were largely senescing (Michaloglou et al., 2005). The detection of senescent cells in early tumoral lesions was then confirmed in many studies under different experimental conditions including oncogenes and types of cancers. Inhibition of key senescence regulators, such as p53 or p21, inhibits senescence and collaborates with oncogenes to form tumors in mice (for review see Collado and Serrano, 2010).

Aside from its link with cancer, cellular senescence has also been proposed since its discovery to promote aging. Indeed, telomere shortening prevents cell immortality, and restoring telomerase activity can immortalize cells (Bodnar et al., 1998). With age and during age-related diseases, the accumulation of senescent cells and its hallmarks, such as p21 and p16 expression, causes decreased telomere length or increased ROS accumulation (for review see de Magalhães, 2004; Smith and Pereira-Smith, 1996; Campisi, 2005). Functionally, decreased telomere length or structure invariably accelerates hallmarks of aging, and the accumulation of senescent cells is probably participating in those phenotypes. In particular, several tissues have a limited renewal capacity (for review see Blasco, 2007; Hao et al., 2005), which may be due to a default in stem cell division. For instance, forcing the division of Sox2+ stem cell populations results in their premature exhaustion. These exhausted cells display features of cellular senescence, which could lead to premature aging (Vilas et al., 2018). Regenerating tissue using induced pluripotent stem cells (iPSCs) is a growing field of research aimed at fighting diseases and limiting aging-related pathologies. The generation of iPSCs from somatic cells is achieved using, for instance, a cocktail of embryonic factors Oct4, Sox2, Klf4, and c-Myc (OSKM), but this strategy has a low efficacy. Cellular senescence is induced during this process by OSKM, likely acting as an oncogene-like signal, and limits cell division and reprogramming (Li et al., 2009; Utikal et al., 2009; Banito et al., 2009).

Hence, for nearly half a century, the effects of cellular senescence were mainly viewed as a consequence of senescence-induced loss of cell proliferation, which can be beneficial, for instance, during tumor initiation, or detrimental, for instance, for tissue regeneration and function during aging (Fig. 1).

## Discovery of the SASP and evolution of a paradigm: From an effect based on loss-of-function to a gain-of-function process

### Discovery of the SASP

**Figure 2.**
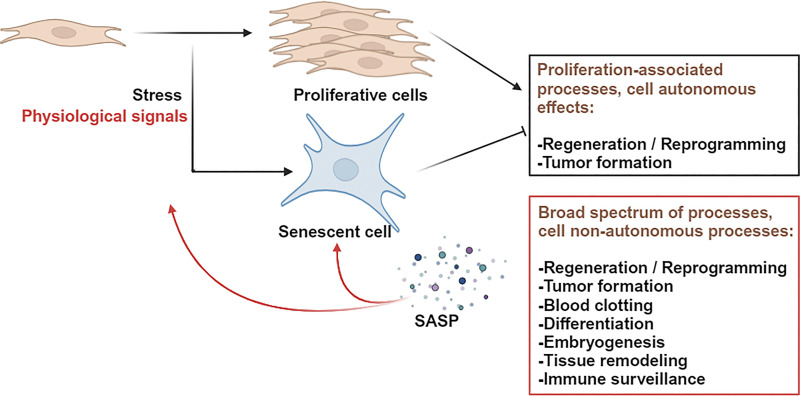
**Change of view in functions of senescent cells with the discovery of SASP.** After its discovery, cellular senescence was viewed as a process stably blocking cell proliferation and thus thought to inhibit proliferation-dependent processes, such as cancer, tissue renewal, or reprogramming (highlighted in black rectangle). After the discovery of the SASP in 2008, the potential effects of senescent cells were no longer limited to cell autonomous effects and to cell proliferation-associated processes. Some examples of the functions of the SASP are mentioned, and they include reinforcement of the senescence phenotype, induction of paracrine senescence (red arrows), and regulation of other processes, highlighted in the red rectangle.

First, it was observed that senescent fibroblasts stimulate the growth of cancer cells via secreted factors (Krtolica et al., 2001). In 2008, this discovery was extended and generalized with the description of the senescence-associated secretory phenotype (SASP). Then the view on the potential effects of senescent cells drastically changed as these were no longer exclusively attributed to an intrinsic proliferation arrest mechanism, but now encompassed an extrinsic influence of the microenvironment (Figs. 1 and 2). The SASP was first described in 2008 by three independent studies. The first detected SASP in fibroblasts under replicative senescence or senescence induced by gamma-irradiation, where senescing cells secreted cytokines, chemokines (e.g., IL6, 8), and growth factors (e.g., GRO and HGF). Similar results were obtained during senescence in epithelial cells and cancer cell lines treated with chemotherapeutic agents and in biopsies of patients with prostate cancer who had undergone chemotherapy (Coppé et al., 2008). The second article showed a rise in the transcription of secreted factors in oncogene RAF-induced senescence of human fibroblasts and melanocytes. The authors reported that IL6 played a central role in the SASP of OIS cells as it acts in both an autocrine and paracrine manner (Kuilman et al., 2008). The third study focused on the IL8 receptor CXCR2 and its ligands, which reinforced senescence in fibroblasts in the context of replicative and oncogene MEK-induced senescence. Upregulation of other secreted factors like IL6 and IL1α was also reported (Acosta et al., 2008). Together these studies demonstrated that the SASP is not only an important hallmark of senescent cells but also a new functional characteristic, changing the effect of senescent cells in the tissue.

### The diversity of the SASP

Initially, studies focusing on the SASP mainly described cytokines and chemokines. An unbiased proteomic analysis of SASP in OIS fibroblasts extended the list of SASP components to several non-inflammatory proteins, such as TGFβ family ligands or VEGF, identifying them as some of the most secreted factors (Acosta et al., 2013). These authors and others also demonstrated that the SASP can induce paracrine senescence and revealed that several secreted factors, for example, TGFβ or IL1, mediate this process, leading to a better understanding of the regulation of paracrine senescence (Cipriano et al., 2011; Acosta et al., 2013). Later, additional master regulators of SASP production were identified. For example, the NOTCH1 receptor suppresses inflammatory SASP while driving a TGFβ-rich secretome in fibroblasts during senescence induced by oncogenic RAS or the chemotherapeutic etoposide. After the induction of senescence, NOTCH1 expression fluctuated, changing the balance between proinflammatory and a TGFβ-enriched SASP. NOTCH1 expression was also found in RAS-driven pancreatic intraepithelial neoplasia (PanIN) of mice (Hoare et al., 2016).

Since then, hundreds of SASP molecules have been identified, and although the first SASP components described were proteins, it is now clear that it is much more diverse, containing lipids, metabolites, ions, and extracellular vesicles that can contain miRNAs and DNA fragments among others (Fafián-Labora and O’Loghlen, 2020). This secretome composition is dynamic and depends on the cell type, the level of senescence-inducing stress, and the time elapsed since the induction of senescence.

### The SASP can have opposite effects to senescence-associated proliferation arrest processes

#### In cancer

Even though the SASP was shown to reinforce and promote senescence, the factors released by a cell into the microenvironment trigger signaling pathways within the same cell and neighboring cells, leading to many potential unexpected functions of the SASP and of senescent cells (Fig. 2). This complexified and diversified the role of senescent cells and led to the discovery that senescent cells can have non-autonomous functions that can exert opposite effects to senescence-associated cell-autonomous, proliferation arrest effects. For instance, if proliferation arrest of senescent cells is implicated in tumor suppression, the SASP of senescent cells was shown both to inhibit, for instance, by inducing paracrine senescence and blocking cell proliferation, or promote cancer. Indeed, in the latter case, the SASP was reported to enhance tumor formation and progression in several tumoral contexts by different mechanisms. First, prolonged p16 expression in the basal epidermis was demonstrated to lead to hyperplasia through the secretion of WNT that stimulates keratinocyte proliferation, leading to hyperplasia and an increase in carcinogen-induced epidermal papillomas. Second, in the same study it was confirmed in human premalignant epidermal lesions, in which some cells expressed p16 (Azazmeh et al., 2020). Third, the SASP was shown to drive hepatic carcinoma development in mice. Indeed, obesity-induced changes in their gut microbiota led to the circulation of a bacterial metabolite, deoxycholic acid, which triggers senescence and SASP production in hepatic stellate cells, and to cancer development following their stimulation with an oncogene (Yoshimoto et al., 2013). Moreover, it was demonstrated that in the pancreas, senescent cells stimulate the growth of premalignant PanIN lesions at least partially through the inflammatory SASP regulator COX2 (Kolodkin-Gal et al., 2022). It was recently shown by two groups that accumulation of senescent macrophages can be observed intratumorally in lung cancer patients. Furthermore, senescent alveolar macrophages were shown to promote KRAS-driven tumorigenesis in mice and senescence inhibition by genetic inactivation of p16 or p21 or senescent cell removal by senolysis impaired tumorigenesis (Haston et al., 2023; Prieto et al., 2023).

It is considered that cell-autonomous effects of senescence, mainly proliferation arrest, are antitumoral, and cell non-autonomous effects, through the SASP, can be protumoral. In most studies linking SASP with cancer development, the effect of senescent surrounding microenvironment cells, such as cancer-associated fibroblasts, is considered. However, there are studies showing that cancer cells undergoing therapy-induced senescence are able to escape and resume proliferation. These escaper cells gained stemness and increased aggressiveness (Milanovic et al., 2018).

#### In regeneration/reprogramming

The SASP of senescent cells was reported to improve cell reprogramming, tissue repair, and renewal. Cellular senescence is induced during wound healing in the skin, and these cells secrete the SASP factor PDGF-AA that induces fibroblast differentiation in myofibroblasts contributing to healing (Demaria et al., 2014). Keratinocytes express a stem cell signature following OIS, which is dependent on the SASP, and this transient SASP induces tissue regeneration and hair growth in engrafted skin in mice (Ritschka et al., 2017). It was also reported that OSKM-driven reprogramming in mice is more efficient in the presence of senescent cells and of IL6 SASP components (Mosteiro et al., 2016), which improve muscle and liver regeneration (Chiche et al., 2017; Cheng et al., 2022). Senescent cells can also be found during embryonic development where they participate in organ patterning and tissue remodeling, possibly via the TGFβ SASP factor (Gibaja et al., 2019; Muñoz-Espín et al., 2013; Storer et al., 2013).

In non-mammalian animals, even more complex structures can be regenerated in adult animals through senescent cells and the SASP. Salamanders regenerate whole limbs, and senescent cells were reported to create a proregenerative environment promoting limb regeneration via the SASP and the WNT pathway (Yun et al., 2015; Yu et al., 2023). Fin amputation in zebrafish leads to the accumulation of senescent cells that contribute to fin regeneration (Da Silva-Álvarez et al., 2020). Remarkably, whole-body regeneration in cnidarians is also regulated by senescence. Indeed, after the removal of the head, senescent cells were observed around the injury site and were indispensable for whole-body regeneration (Salinas-Saavedra et al., 2023). We speculate that the SASP is likely involved in these last two examples.

#### The ever-growing list of paracrine SASP effects

Below, we present only a few examples of the literature to illustrate the diversity of the pathophysiological effects of senescent cells through their secretory phenotype. One such effect is the promotion of cell differentiation. Conditioned media of senescent cells induced by mitochondrial dysfunction inhibits adipogenesis of preadipocytes and promotes keratinocyte differentiation (Wiley et al., 2016). SASP-dependent fibroblast-to-myofibroblast differentiation in the lung was also reported. Using an organoid-based lung fibrosis model, authors demonstrated that cellular senescence in alveolar epithelial AT2 cells activates SASP components, including TGFβ that reinforces the senescence phenotype of AT2 cells through a positive autocrine loop and promotes differentiation from fibroblast to myofibroblast via a paracrine effect. The authors speculated that this fibroblast differentiation induced by the SASP of epithelial senescent cells could be responsible for idiopathic pulmonary fibrosis (IPF) (Enomoto et al., 2023). An unbiased proteomic approach on conditioned medium from human foreskin fibroblasts highlighted new SASP factors involved in hemostasis. This study reported that the SASP of senescent fibroblasts increases the number of platelets and enhances their activity (increase of ATP release), thus reducing blood clotting time (Wiley et al., 2019). Unsurprisingly, the SASP has many immunemodulatory activities. For instance, pre-malignant senescent hepatocytes secrete various chemokines and cytokines that trigger the recruitment of immune cells such as CD4 T lymphocytes, monocytes, and macrophages which, in turn, lead to an immune response and the clearance of senescent hepatocytes (Kang et al., 2011). Inversely, it was shown that in the long term, the SASP of senescent cells displays immune suppressive properties that lead to the inhibition of “senescence immunosurveillance” (Toso et al., 2014; Eggert et al., 2016). Senescent cells may also exert more specific functions via their SASP. It is well-known that cellular senescence and natural killer (NK) cells contribute to liver pathologies. In one study, the authors suggested that senescent hepatocytes through the SASP, including CXCL-10, enhance NK cell migration in vitro through the CXCR3/CXCL-10 axis, and their cytotoxic activity by increasing the expression of IFNγ and CD107a (a marker of NK cell activation) (Zang et al., 2019).

In conclusion, senescent cells can have complex cell autonomous and non-cell autonomous effects through their SASP (Fig. 2), affecting both themselves and neighboring cells. Nevertheless, little is known on whether reinforcement, establishment, and change in functions and/or identity induced by senescent cells can be associated with the senescent phenotype, in other words whether senescent cells can display a gain/modification (reinforcement, establishment and change) of their function and/or identity.

## Senescent cells can display reinforcement, establishment, and change in their function and identity

### Cellular senescence promotes the reinforcement of cell function and identity

**Figure 3.**
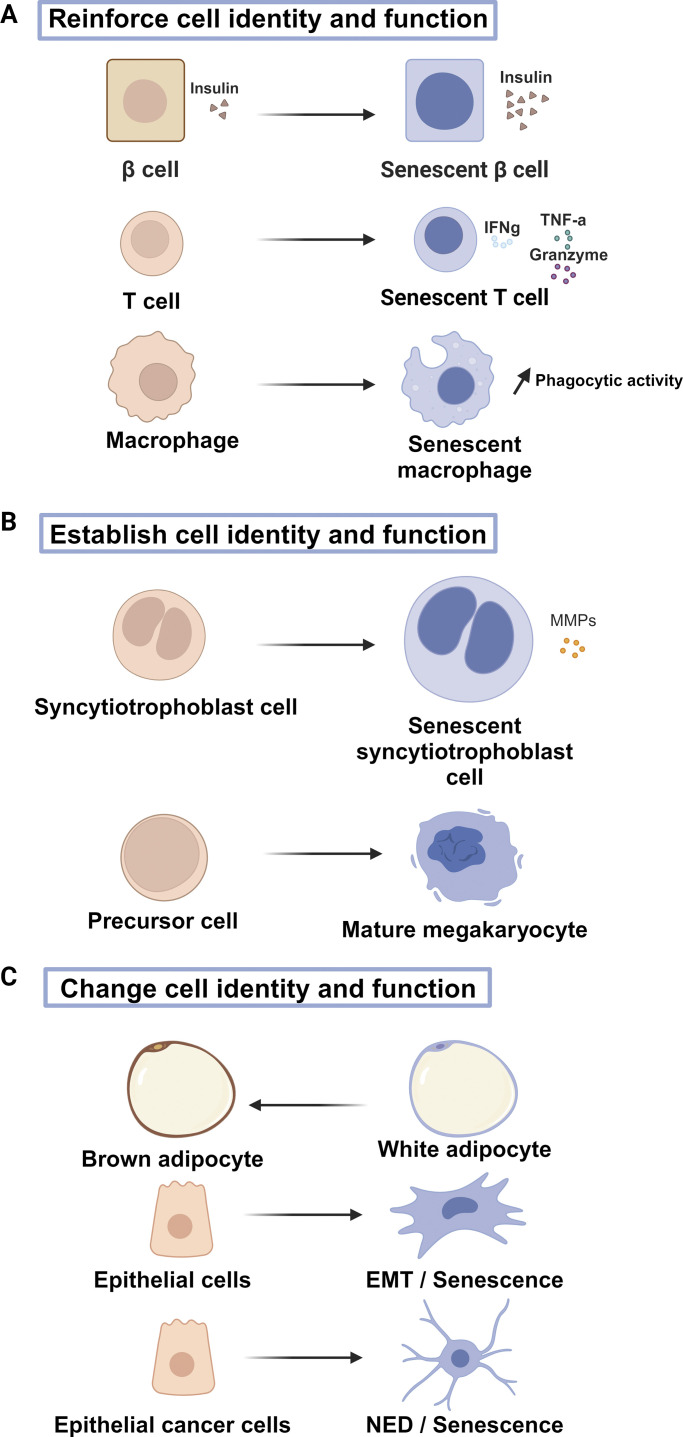
**Senescent cells can display reinforcement, establishment, and change in their function and identity. (A)** Differentiated senescent cells can display a reinforced identity and function: senescent β cells secrete more insulin; senescent T cells produce more IFNγ, TNFα, and granzymes; senescent macrophages display increased phagocytic activity. **(B)** Entry into senescence can contribute to establishing cell identity and cell function as shown here for maturation of syncytiotrophoblasts and production of mature megakaryocytes. **(C)** Senescence can change cell function and identity: adipocytes can change their properties depending on whether they are senescing or not, and epithelial cells can display transdifferentiation when they are senescing (EMT has been reported in epithelial non-tumoral cells and NED reported in epithelial hormone-dependent cancer cells).

The discovery of the SASP and additional markers of senescence has led to the detection of senescent cells in differentiated, non-proliferating cells, and description of changes in their function. Indeed, beyond the classical effects of senescent cells (cell autonomous loss-of- proliferation and communication through the SASP to the environment), they were shown to reinforce their function and identity under some circumstances (Fig. 3 A).

For instance, senescent pancreatic beta cells display increased insulin production, which impacts type I diabetes in a mouse model. By increasing deep senescence regulator p16, beta cells were driven into senescence. These senescent beta cells displayed increased glucose-stimulated insulin (GSIS) secretion. This process, at least initially, improved glucose homeostasis in mice. The authors showed that p16 enhanced GSIS through CDK4 inhibition, and that p16 induction in diabetic mice improved glucose tolerance. They also showed that an increase in endogenous, age-associated p16 improves beta cell maturity in humans and mice. This mechanism may partially decrease insulin sensitivity in tissue (Helman et al., 2016).

Highly differentiated effector T cells that re-express CD45RA, a typical marker of naïve T cells (T EMRA), are considered the end stage of T cell senescence. They exhibit short telomeres and a decrease in proliferation. EMRA cells have increased KLRG1, γH2AX, and CD57, and decreased Ki67. Interestingly, they produce more perforin and granzyme cytotoxic proteins and secrete higher levels of inflammatory molecules (IFN-γ and TNF-α), supporting their increased function upon cellular senescence (Henson et al., 2015).

By using tomato knock-in mice at the p16 locus, to follow p16-positive cells in vivo at the single cell level, it was demonstrated that p16-positive macrophages displaying senescence features (e.g., cell cycle arrest, positive for SA-β-Gal and expressing SASP factors) have an increased phagocytic activity (Liu et al., 2019).

This increase in cell function and identity may only be transitory, and we can speculate that it could become deleterious in the long term.

### Senescence promotes the establishment of cell function and identity

In addition to reinforcing cell identity and function, senescent cells can also play a role in the establishment of cell identity and function (Fig. 3 B).

For instance, cellular senescence contributes to the formation and function of the multinucleated syncytiotrophoblast layer, which is needed for fetal feeding during gestation. In one study, analysis of placentas from pregnancies with intrauterine growth restriction pathology, which is characterized by reduced fetal development, showed a decrease in senescence markers. In mice negative for p16 and p21, placentas had disrupted tissue architecture and showed hyperplasia. Authors speculated that the increased cell size of senescent syncytiotrophoblast cells facilitates the exchange of nutrients between the mother and embryo because of the larger surface area. Interestingly, in accordance with the hypersecretory phenotype, these senescent cells have increased expression of matrix metalloproteases, which are necessary for the syncytiotrophoblast invasion (Chuprin et al., 2013; Gal et al., 2019).

Senescence appears to be a normal part of megakaryocytic maturation. Mature megakaryocytes differentiate from precursor cells in response to a non-inflammatory cytokine thrombopoietin (TPO). Megakaryocyte cell lines exposed to TPO first proliferate, then enter irreversibly into cell proliferation arrest and show positivity for p21, p27, SA-β-Gal, and inflammatory SASP gene expression. This TPO-induced senescence was shown to be MAPK/RAS/ERK-dependent and to rely on p21 but not on p53, p16, and p27 activity. Cell sorting and analysis from the human bone marrow showed that senescence is also present in mature megakaryocytes in vivo (Besancenot et al., 2010). In contradiction to this, it has been demonstrated that senescence in macrophages isolated from the spleen of mice treated with ionic irradiation display reduced phagocytosis, which was restored by the elimination of p16 expression (Palacio et al., 2019). These differences in senescence effect on macrophage function could be explained either by different types of senescence: replicative senescence versus ionic radiation-induced or by the timing, in which case senescence could first induce hyperfunction followed by exhaustion and loss of function.

### Cellular senescence can change cell function and identity

Aside from reinforcing or establishing cell function and identity, senescence can also contribute to altering these features in some contexts. Senescent cells have been demonstrated to change cell identity in a cell-autonomous or non-autonomous manner (Fig. 3 C).

Cellular senescence can regulate cell differentiation toward white fat cells. White adipocytes store excess energy as triacylglycerol, while brown adipocytes are involved in thermoregulation. Mice harboring a knockout for the major senescence regulatory gene *Cdkn2a* displayed increased expression of brown fat cell markers in inguinal white adipose tissue. The authors further showed that induced pluripotent human cells, which can differentiate into adipose precursors, display higher brown cell marker expression in CDKN2A knockdown condition. This shows that senescent cells can change the properties and function of adipocytes (Rabhi et al., 2018).

Cellular senescence can also drive the expression of lineage-inappropriate genes that could modify or perturb cell identity. For example, Late Cornified Envelope LCE2, a gene expressed in terminally differentiated keratinocytes, is derepressed in senescent fibroblasts. Expression of this marker in terminally differentiated keratinocytes in senescent fibroblasts is due to decompaction of the locus and activation of p53 and C/EBPβ signaling (Tomimatsu et al., 2022).

Remarkably, in some conditions, senescent cells undergo more advanced identity changes. For example, the SASP of senescent cells promotes neuroendocrine transdifferentiation (NED) of breast and prostate hormone-dependent cancer cells. Indeed, conditioned medium from senescent fibroblasts induces paracrine senescence and growth of neurite-like ramifications and neuroendocrine marker SCG2 and CHGB expression in MCF7 breast cancer cells supporting that senescent MCF7 cells display NED. In the same study, similar results were obtained in senescent LNCaP prostate cancer epithelial cells. Of note, breast and prostate non-hormone-dependent cancer cells do not enter into senescence and NED when exposed to the SASP. Nevertheless, co-occurrence of senescence and NED and whether it is restricted or not to hormone-dependent cells will have to be confirmed in different cellular models, and whether there are some interdependencies between these two phenotypes will need to be investigated (Raynard et al., 2022).

The SASP can also promote an epithelial-mesenchymal transition (EMT) in a paracrine manner in neighboring cells (Coppé et al., 2008). Changes from an epithelial to mesenchymal morphology, even if partial, are dramatic and impact cell fate by contributing to physiological and pathological processes. EMT is involved in embryonic development and wound healing in a physiological context or in cancer in a pathological context, as it is considered a driving force for migration, invasion, and tumor metastasis. Conditioned medium from senescent fibroblasts induces cell scattering in several non-invasive epithelial breast cancer cell lines. SASP induced a decrease in epithelial cell surface proteins, such as E-cadherin and β-catenin, an increase in mesenchymal protein vimentin, and a relocation of Claudin-1 from the membrane to the nucleus (Coppé et al., 2008). Interestingly, in more recent studies, it was shown that during epithelial cell senescence, cells can also exhibit marks of EMT. In one study, they discovered that OIS in epithelial cells decreased cell contact and increased their expression of EMT markers such as fibronectin, vimentin, TGFβ factors, and/or embryonic factors (such as SNAI, ZEB1) according to transcriptomic data analysis (dataset GSE110884 in Warnier et al., 2018). Consistently, MCF-7 epithelial breast cancer cells undergoing chemotherapy-induced senescence showed significant enrichment in the EMT gene signature through transcriptomic analysis (Czarnecka-Herok et al., 2022). These last two studies suggest that EMT and senescence co-occur in the same cell population. More strikingly, normal mammary epithelial cells were confirmed to exhibit both senescence (decrease in cell proliferation, Ki67, SA-β-gal positivity, and expression of SASP genes) and EMT features (EMT gene expression, vimentin and fibronectin increase, and E-Cadherin decrease in the protein level) in response to TGFβ (Huna et al., 2023). In addition, human foreskin keratinocytes, cultivated without a feeder cell layer, were shown to undergo senescence, as illustrated by an increase in p16 and a decrease in cell proliferation, and concomitantly loss of some epithelial markers, such as desmosome components and epithelial keratins, as well as increased expression of the classical EMT genes vimentin and fibronectin (Darbro et al., 2005).

Spontaneous escape of the proliferation arrest during oncogene-induced senescence was observed in normal bronchial epithelial cells and genomic instability gained during the senescence is responsible for this escape. Escaping cells displayed epithelial–mesenchymal transition features, such as increased invasiveness (Zampetidis et al., 2021). We can speculate that some EMT features were already there in senescent cells and that cells which escaped the proliferation arrest kept these features.

Despite some examples suggesting that senescent epithelial cells display EMT features, the generalization of this observation of EMT in epithelial senescent cells through single-cell studies to show their co-occurrence in the same cells, and functionally linking them at both the cell-autonomous and non-autonomous levels is currently lacking.

## Conclusions and perspectives

During the 60 years of research on cellular senescence, our understanding of the biology and complexity of senescent cells has progressively improved. If cellular senescence was initially considered a stable proliferation arrest with a tumor suppressive function and detrimental effect on tissue regeneration, the discovery and characterization of the SASP revealed different and opposite effects of senescent cells on their neighboring environment. In more recent years, it became clear that senescent cells can undergo dramatic changes in function and identity. In some cases, as illustrated above, senescent cells display reinforcement, establishment, or change in their cell function and identity. Nevertheless, we are only at the dawn of research in this field, and the functional connection between the senescent status/hallmarks (proliferation, SASP, and potentially others) and gain-of-function or change in cell identify will have to be investigated. The consequence of such gain- or change-of-function of senescent cells may offer new perspectives to better apprehend the role of senescent cells in pathophysiological settings. We would also like to mention that a persisting limit in interpreting the results in cellular senescence field is that no single factor allows to identify senescent cells or allows to impact specifically and only senescent cells, always limiting the interpretation of the results.
